# Validation of post‐traumatic growth inventory in mothers with the experience of having the NICU‐Hospitalized newborns “validation of post‐traumatic growth inventory”

**DOI:** 10.1002/nop2.1779

**Published:** 2023-05-05

**Authors:** Nahid Bayrami, Maryam Rassouli, Azam Shirinabadi Farahani, Mehdi Heidarzadeh, Fatemeh Khademi, Salehe Tajlli, Mohadese Babaie, Anahita Masoum poor, Khadijeh Hatamipour

**Affiliations:** ^1^ Department of Pediatrics and Neonatal Intensive Care, School of Nursing and Midwifery Shahid Beheshti University of Medical Sciences Tehran Iran; ^2^ Cancer Research Center, School of Nursing & Midwifery Shahid Beheshti University of Medical Sciences Tehran Iran; ^3^ Department of Pediatric & Neonatal Intensive Care Nursing, School of Nursing & Midwifery Shahid Beheshti University of Medical Sciences Tehran Iran; ^4^ Department of Nursing, School of Nursing and Midwifery Ardabil University of Medical Sciences Ardabil Iran; ^5^ Department of Nursing, School of Nursing & Midwifery Arak University of Medical Sciences Arak Iran; ^6^ School of Nursing and Midwifery Tehran University of Medical Sciences Tehran Iran; ^7^ Department of Nursing, Tonekabon Branch Islamic Azad University Tonekabon Iran

**Keywords:** mothers, neonatal intensive care unit, newborn, post‐traumatic growth, reliability, tool development, validity

## Abstract

**Aims:**

Investigating post‐traumatic growth (PTG) in mothers with the experience of having a preterm newborn hospitalized in the NICU requires a valid tool. This study aims to determine the validity and reliability of the Farsi version of the post‐traumatic growth inventory (PTGI) in mothers with the experience of having their newborns hospitalized in the NICU.

**Design:**

This study was methodological research.

**Methods:**

In this study, 250 mothers who had newborns with a history of NICU hospitalization during the last 3 to 12 months and had visited paediatric clinics of the selected hospitals in Tehran with the aim of having their children's condition examined were selected through convenience sampling. The data were collected using a demographic information questionnaire and PTGI. The face validity, the construct validity (confirmatory factor analysis), and the internal consistency reliability of the inventory were measured using SPSS V22 and LISREL V8.8.

**Results:**

According to appropriate values for factor analysis fit indices (FI = 0.94, RMSEA = 0.07, IFI = 0.94, NFI = 0.93, RFI = 0.91, NNFI = 0.93, SRMR = 0.07), 21 items and 5 factors were confirmed for this inventory. Furthermore, Cronbach's alpha coefficient of this inventory was measured as *α* = 0.94.

**Conclusion:**

According to favourable psychometric properties, the Farsi version of PTGI is a suitable tool for studying PTG in mothers with the experience of having preterm newborns in the NICU. Using PTGI can help nurses in planning family‐centered care interventions to reduce the impact of the mental trauma caused by the preterm newborn's hospitalization in parents.

**Patient or Public Contribution:**

Mothers who had newborns with a history of NICU hospitalization during the last 3–12 months.

## 1 INTRODUCTION

Preterm birth and the subsequent NICU hospitalization is a stressful and worrying experiences for parents and create a situational crisis (Aftyka, Rozalska‐Walaszek, et al., 2017), while confronting the mother with traumas of separation and numerous challenges caused by the disease outcome, the treatment procedure, and care (Dhanoa & Singh, 2021).

Under these circumstances, mothers experience high levels of stress and statistically significant feelings of hopelessness, and this process overshadows the emotional and physical development of the baby and the mother‐infant interaction. However, there is evidence that the process of coping with these challenges may lead to positive changes in parents. Much evidence has focused on a phenomenon called Post Traumatic Growth (PTG) in recent years (Aftyka et al., 2020; Aftyka, Rybojad, et al., 2017; Wilson & Cook, 2018).

PTG refers to positive psychological changes that are the result of coping with great challenges and difficult life events (Tedeschi & Calhoun, 1996). In other words, positive PTG reflects the caregiver's mental ability to perform protective and regulatory functions in traumatic situations (Aftyka, Rybojad, et al., 2017). In this regard, Boztepe et al. (2015) believe that mothers who have experienced the hospitalization of their newborns in the NICU achieve some degree of PTG (Boztepe et al., 2015). Instead of engaging in threats, horrific memories, or negative emotions caused by newborns' hospitalization, these mothers focus on providing care for the newborn (Aftyka, Rybojad, et al., 2017) and keeping their spirits high, maintaining their resilience, and have a good performance in regard with the newborn and its needs, despite experiencing a different form of motherhood (Wilson & Cook, 2018).

According to the cognitive theory of Boztepe et al. (2015), new thoughts in those who experience a stressful incident can be the source of a positive change in them. Therefore, measuring PTG and its dimensions can help nurses as professional experts who have a close relationship with mothers use positive thoughts as a strategy for mothers to cope with stressful situations while inducing these thoughts in them, and also help them overcome the problems caused by the stressful incident to create an opportunity for growth out of the negative effects of this situation (Boztepe et al., 2015). Despite the importance of this issue, few studies are done in this field and most research focuses on a population of patients with terminal illnesses, such as cancer, and their families (Heidarzadeh et al., 2017; Morris et al., 2013). Therefore, investigating PTG in mothers who have experienced the hospitalization of their newborns in the NICU and, consequently, the need to measure this concept is an undeniable necessity.

Certain tools have been introduced to measure PTG (Tedeschi & Calhoun, 1996; Walsh et al., 2018). In 1996, Tedeschi & Calhoun developed a 21‐item tool under the title Post Traumatic Growth Inventory (PTGI) to measure PTG in adults, which examines mental development in a variety of dimensions, even during the most challenging events of life (Tedeschi & Calhoun, 1996). So far, this tool has been translated into different cultures and in different languages (Aydin & Kabukçuoğlu, 2020; Pajón et al., 2020; Shirinabadi Farahani et al., 2021; Silva et al., 2018). Its validation has also been done in different countries and it has been introduced as a valid and reliable tool for many population groups (García & Wlodarczyk, 2016; Silva et al., 2018). The Farsi version of this tool has been validated by Heidarzadeh et al. (2017) in Iran, among adult patients with cancer (Heidarzadeh et al., 2017).

This study is done with the aim of determining the reliability and the validity of PTGI in the mothers with the experience of having a newborn hospitalized in the NICU and measuring their PTG, given the fact that PTG depends on the social and cultural background of the population under research (Heidarzadeh et al., 2018) and considering the positive and constructive impact of PTG on mothers' performance, helping them play their role in providing care for the newborn, and the need for a suitable tool to assess this concept and to determine the effectiveness of interventions designed and implemented for improving PTG in mothers.

## 2 METHODS

This methodological study was conducted from November 2018 to August 2019 in the teaching hospitals affiliated to Shahid Beheshti University of Medical Sciences in Tehran, Iran.

The research samples consisted of the mothers whose newborns had been hospitalized in the NICUs of the hospitals affiliated to Shahid Beheshti University of Medical Sciences 3–12 months prior to the study. Furthermore, the samples had no history of psychiatric diseases and had experienced no other stressful event other than the aforementioned incident during the determined period. The samples were selected through convenience sampling during visitations and their presence in clinics. To this end, after the visitation, the mothers were provided with the questionnaires in the waiting room. Filling out each questionnaire took approximately 15–30 min.

Data collection in this research was done using two questionnaires. First, the demographic and clinical information questionnaire including items regarding age, the level of education, the mother's marital status, birth order, the adequacy of family income, the level of support provided by relatives, the number of children in the family and the items about the time passed after the newborn's hospitalization and the demographic and clinical status of the newborn including gestational age, the type of the disease at the time of birth and the newborn's current health status. The second tool, PTGI, consisted of 21 items. This tool was translated into Farsi and validated among people with cancer by Heidarzadeh et al. (2017) which determines five areas of psychological growth after a stressful incident including *identifying new opportunities*, *communication with others*, *becoming stronger*, *appreciation of life* and *spiritual changes* (Heidarzadeh et al., 2017). This tool is scored on a 6‐point Likert scale: 0 (generally none), 1 (to a very low extent), 2 (to a low extent), 3 (to a moderate extent), 4 (to a high extent) and 5 (to a very high extent). The higher the score, the higher the PTG and vice versa. In the present study, in order to evaluate the psychometric properties of the tool in the mothers with newborns hospitalized in the NICU, the face validity, the construct validity and the internal consistency were measured. For the measurement of face validity and cognitive evaluation, during face‐to‐face interviews, 10 mothers meeting the inclusion criteria were asked to express their opinions on the ease of use and the understandability of sentences and phrases or any possible ambiguity in the meanings of words (Keeley et al., 2013). To determine the construct validity and confirm PTGI dimensions, confirmatory factor analysis (CFA) was performed, according to which a sample size of 200 subjects was recommended based on the factors (Kline, 2015). Accordingly, and taking into account the sample drop‐out, a total of 250 mothers were included in the study. CFA is a technique used to examine the fit between the hypothetical model and the data obtained from research samples. To evaluate the fit of the model, the maximum likelihood algorithm was used. There are numerous fit indices to test the appropriateness of the model and it is recommended to use several of them (Kline, 2013). To determine the internal consistency reliability, Cronbach's alpha coefficient was calculated using the data of all the participants.

The present study is approved by the ethics code IR.SBMU.PHARMACY.REC.1397.021. Before distributing the questionnaires, the researcher obtained informed consent from mothers, gave them full explanation regarding the questionnaire and assured them of the confidentiality of the data.

After being filled out, the questionnaires were collected and analysed using SPSS V22 and LISREL V8.8. The goodness of fit indices were used to confirm the dimensions, including chi‐square, comparative fit index (CFI) and incremental fit index (IFI). Cronbach's alpha was also calculated to determine the internal consistency. The ordinal and categorical data and absolute and relative frequencies were calculated for the descriptive analysis of quantitative variables, mean and standard deviation.

## 3 RESULTS

The data were analysed using 250 questionnaires filled out by the mothers with experience of having NICU hospitalized newborns. The mean age of the mothers was 30.24 years; the mean gestational age at birth, 33.02 weeks; the mean birth weight of newborns, 1876.77 g; and the current age of the child, 76.7 months. Other demographic and clinical features can be seen in Table 1.

**TABLE 1:** Demographic and clinical characteristics of study participants.

	Variables	*N* (%)
Number of children in the family	One	122 (49/4)
	Two	103 (41/7)
	Three	20 (8/1)
	More than three	2 (0/8)
Birth order	First	125 (50/2)
	Center	1 (0/4)
	End	123 (49/4)
Level of education	Primary	8 (3/2)
	Secondary	112 (40/9)
	University	129 (51/8)
Employment status	Housewife	175 (70/6)
	OFFICIAL	54 (21/8)
	Free	19 (7/7)
Adequacy of family income	Low	89 (35/6)
	Medium	154 (61/6)
	Very	7 (2/8)
Level of support provided by relatives	Low	53 (21/4)
	Medium	146 (58/9)
	Very	49 (19/8)
Newborn's current health status	Severe illness	25 (10)
	Mild illness	43 (17/3)
	Only immature	161 (64/7)
	Mild illness and immaturity	17 (6/8)
	Severe illness and prematurity	3 (1/2)

The results of the cognitive evaluation of the questionnaire through interviews with mothers showed that none of the expressions needed any corrections. In order to investigate the factor structure of PTGI in the mothers with the experience of having newborns hospitalized in the NICU, CFA was performed and a 5‐factor model was examined. The fit indices of model are displayed in Table 2, along with the acceptable values of indices.

**TABLE 2:** Confirmatory factor analysis fit indices in 5‐factor model (with 21 items).

Model	*χ* ^2^	*df*	*χ* ^2^/*df*	CFI	IFI	RMSEA	NFI	NNFI	RFI	SRMR
5 factors (with 21 items)	921.52	185	4.98	0.94	0.94	0.07	0.93	0.93	0.91	0.07
Acceptable values	–	–	1–5	≤0.9	≤0.9	≥0.1	≤0.9	≤0.9	≤0.6	≥0.05

**FIGURE 1:**
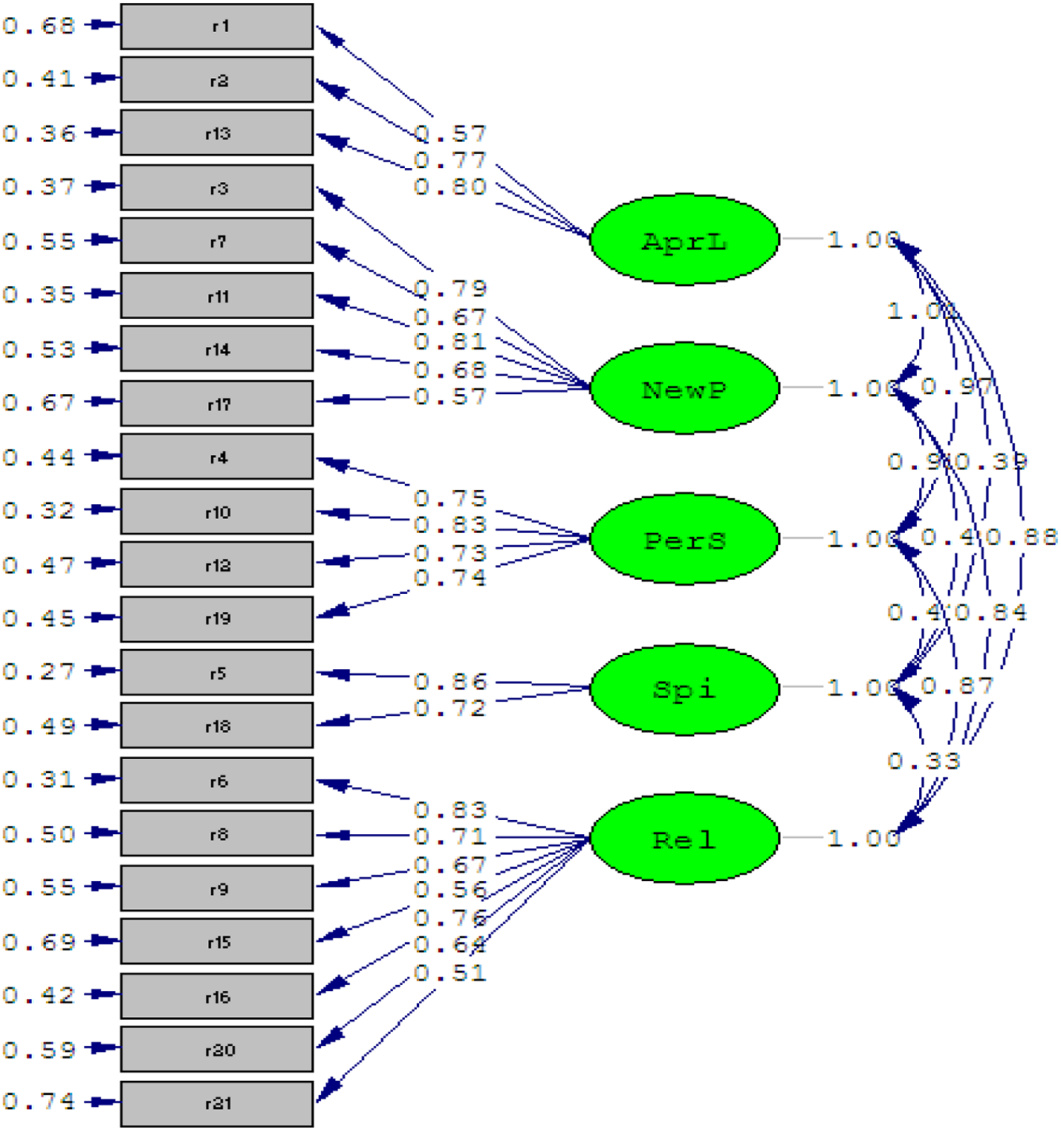
Standardized model of inventory dimensions and its relationship with inventory items and main structure.

According to Figure 1, the results of CFA for the 5‐factor model show that all dimensions have a favourable correlation coefficient with the related items. In addition, according to the T‐value test performed by LISREL, all the correlations between dimensions and their items are statistically significant and there is no inconsistency. In general, according to the obtained indicators (Table 2), it can be said that the model and its constituent concepts are acceptable and the PTGI is approved with 21 items and 5 factors in the mothers with the experience of having newborns hospitalized in the NICU. Moreover, the internal consistency of the tool was examined and the Cronbach's alpha was calculated to be 0.94 (Table 3).

**TABLE 3:** Cronbach's alpha in the post‐traumatic growth inventory and its dimensions.

Subscale	Cronbach's alpha	Number of items
Identifying new opportunities	0.82	5
Communication with others	0.85	7
Becoming stronger	0.84	4
Appreciation of life	0.77	3
Spiritual changes	0.76	2
Total	0.94	21

The mean score of PTG in the mothers who had NICU hospitalized newborns was 72.58 ± 14.19. The mean PTGI scores in each of the dimensions including *identifying new opportunities*, *communication with others*, *becoming stronger*, *appreciation of life* and *spiritual changes* can be seen in Table 4.

**TABLE 4:** Mean score of post‐traumatic growth inventory dimensions in mothers with neonatal experience in NICU.

Dimensions	Mean	Standard deviation	Maximum	Minimum	Score in the inventory	Mean of the item
Identifying new opportunities	17.46	3.98	25	5	0–25	3.49
Communication with others	23.18	5.25	35	6	0–35	3.31
Becoming stronger	12.88	3.36	20	3	0–20	3.22
Appreciation of life	11.16	2.25	15	3	0–15	3.72
Spiritual changes	7.79	1.77	10	1	0–10	3.89
Total score	72.58	14.19	105	33	0–150	

## 4 DISCUSSION

Although giving birth to a premature baby and its being hospitalized in the NICU is an unpleasant experience and a stressful situation for parents, especially mothers, new experiences and positive changes in the mental functioning of these mothers may occur, known as post traumatic growth (Aftyka et al., 2020). The aim of this study was to determine the validity and the reliability of the Farsi version of PTGI in the mothers with the experience of having a preterm newborn hospitalized in the NICU and measuring PTG. The results of the structural equation model in this study showed that the 5‐factor PTGI model in the mothers with the experience of having newborns in the NICU has appropriate goodness of fit indices and is approved.

Reviewing numerous studies related to the factor structure of PTGI shows its diversity among different populations. For instance, the 5‐factor model of the tool is confirmed in the American, Portuguese, Brazilian, Turkish, French, Pakistani versions and recent Iranian studies (Aslam & Kamal, 2019; Aydin & Kabukçuoğlu, 2020; Cadell et al., 2015; García & Wlodarczyk, 2016; Heidarzadeh et al., 2018; Lamela et al., 2014; Palmer et al., 2012; Silva et al., 2018), and the 4‐factor model, in the Japanese and the Chinese versions (Ho et al., 2004; Taku et al., 2008).

But the results of PTGI psychometric studies in Spain over different years and in different populations were statistically significant and challenging. A 2018 study by Pérez San Gregorio et al approved a five‐factor structure similar to the original version of the tool among liver transplant recipients (Perez‐San‐Gregorio et al., 2018). A recent study on Hispanic adults who were the victims of childhood violence reported a 4‐factor structure (Pajón et al., 2020). However, in two other studies done in 2017 and 2016, the 3‐factor version has been confirmed. The research population in the first study consisted of the adults with HIV (Rodriguez Rey et al., 2016) and in the other, the parents whose children had been hospitalized in the ICU due to life‐threatening conditions and survived (Rodriguez Rey et al., 2016). This variation in the dimensions of PTG among the Spanish population despite the common language and culture can be associated with the differences and variations in participants and the experienced stress. It should also be noted that the validation of this tool with such limited clinical samples reduces the generalizability of its results.

In different studies, different structures of PTGI are offered compared to the original version. This difference in factor structures can be explained by different sample sizes and the fact that data may be obtained from a small sample size (Aslam & Kamal, 2019).In addition, there may be translation errors because it is not easy to find equivalents to some words and translate them accurately into another language to maintain the original meaning. Furthermore, it should be noted that these studies have been conducted on different populations who had experienced a range of totally different stressors, such as having cancer or having children with cancer or other illnesses. This difference indicates that stress intensity plays an important role in PTG and its dimensions. In addition to all the important and statistically significant factors that were mentioned, the differences in the cultural background of various populations and the diversity of subjects' religious views and beliefs should not be ignored.

The only study that confirmed the 6‐factor version of this tool was conducted by Morris et al. (2013), which, in addition to the 5 dimensions in the tool, contains the dimension *compassion* accounting for half the variance in principal component analysis (PCA) (Morris et al., 2013). The main tool has an item related to compassion which belongs to dimension *communication with others*. However, in this study, following a qualitative study conducted by the same authors, new items have been added to the tool that examine the feelings and behavioural aspects of people with cancer in the form of a new dimension. Its convergent validity showed that the new items of this dimension fully assess the concept of compassion. Additionally, the results of the psychometric evaluation of PTGI among the patients with prostate cancer approved the items of the dimension *compassion*. The authors believed that showing compassion for others allows cancer survivors to go through the tragic outcomes of the diagnosis and treatment, to expand their views of themselves and their role in the world and to help others in similar situations.

Although the theoretical form and content of dimensions are maintained despite the structural differences in the number of dimensions and items, a completely different structure of PTGI is observed in the Chinese version. In fact, in this version, PTG is introduced as a 4‐factor structure including *self*, *spiritual*, *life orientation* and *interpersonal* (Cheng et al., 2017). The cultural differences seem to be a reason for the diversity of people's responses and reactions to adversity, which in turn has led to a structural diversity in the tool, as culture is a very important variable affecting the dimensions of growth among different populations (Aslam & Kamal, 2019; Heidarzadeh et al., 2017). In addition, the role of the nature of adverse incidents and different stressful situations investigated by these studies cannot be ignored. In other words, the structural diversity created in this tool can be explained by the differences in the coping styles and the strategies of individuals in the face of different stressful events that are influenced by the cultural context of different societies.

Despite the numerous studies on the tool and its translated versions offering acceptable factor structures, in some cases, the initial model does not fit the data obtained from the research population and the theoretical model is not confirmed. Therefore, it would be necessary to develop a new tool appropriate for each specific population. For example, in a 2021 study by Shirinabadi Farahani et al. (2021), according to the goodness of fit indices, neither the 5‐factor model, nor any other models were approved, showing differences in PTG between this specific population and other research populations (Shirinabadi Farahani et al., 2021), which can be due to the fact that the participants were the children with cancer who had a completely different understanding of this concept. In addition, Iran consists of a population with diverse ethnicities, religions and customs resulting in the emergence of various subcultures, which in turn creates different types of lifestyle. All these factors which influence one's level of resilience of PTG are considered as other reasons for rejecting the model among this population. Even after performing exploratory factor analysis and proposing a 3‐factor model and removing the two other items, the two factors *new possibilities* and *personal strength* were not approved. The results of another study by Osei‐Bonsu et al. (2012) also showed that the factor structure of PTGI is unclear among those with the experience of DSM‐IV defined traumatic events (Osei‐Bonsu et al., 2012).

Regarding the other results obtained in the present study, Cronbach's alpha coefficients for the tool and its dimensions were high, similar to the original study where the tool was developed (Tedeschi & Calhoun, 1996) and the study by Heidarzadeh et al. (2017) that has validated the tool in Iran (Heidarzadeh et al., 2017)، This indicates the consistency of the items and the stability of the dimensions. This coefficient has also been reported as acceptable in similar studies (Aftyka et al., 2020; Brelsford et al., 2020).

The mean score of PTG for the subjects was reported to be high, similar to those obtained in the studies of Aftyka et al. (2020), Boztepe et al. (2015), Brelsford et al. (2020), and Byra et al. (2021). Although the mothers have experienced high PTG, they have grown distinctly in different dimensions. For instance, in line with the results of two other studies including Aflakseir and Manafi (2018) and Palmer et al. (2012), the highest and lowest mean scores were assigned to the dimensions *communication with others* and *spiritual changes*, respectively. Similar results were obtained in a study that investigated PTG in the mothers of the children with cystic fibrosis and their coping strategies. In other words, in these mothers, the least amount of growth has occurred at the spiritual level compared to the useful changes observed in communication with others (Byra et al., 2021). However, a Polish study examining PTG in the parents of the newborns hospitalized in the NICU yielded different results (Aftyka et al., 2020). In addition, the scores of spiritual changes in the subjects were high in the studies conducted by Aydin and Kabukçuoğlu (2020), Aslam and Kamal (2019) and Heidarzadeh et al. (2017), compared to the other dimensions of PTG. In fact, the subjects' growth in the spiritual dimension varied depending on factors such as religious beliefs, because the studied population in these countries often have religious beliefs, which in turn leads to these results. The study by Brelsford et al. (2020) examined the relationship between PTG and spirituality in parents after their infants were discharged from the NICU. The results show that parents regarded the parent–child relationship as sacred. In addition, the parents with higher scores in *positive forms of religious coping* and *open spiritual disclosure with spouse* experienced higher levels of PTG even with severe stress (Brelsford et al., 2020). The inconsistency in the results of studies in regard with these dimensions reflects the importance of social, cultural and religious beliefs among the research population, because culture plays an important role in forming beliefs, determining the approaches to understanding stressful situations and positive progress during a critical condition. However, in line with the present study, numerous other studies indicate that most participants have experienced less growth and changes in spiritual issues after going through a stressful situation (Byra et al., 2021; Cadell et al., 2015; Pajón et al., 2020). Because psychologically, one does a reappraisal of the situation to cope with a stressful situation, which is influenced by the level of stress, personality development, spiritual beliefs or reprioritization of important concerns in one's life which is a predictor of PTG and its dimensions (Barr, 2011).

A high level of PTG and all the positive changes and intrapersonal growth that follow depend on the type of the adopted coping strategy (Byra et al., 2021). For example, it can be said that the dimension *communication with others* is related to the social support perceived by parents (Li et al., 2012). One of the coping strategies that mothers adopt in the face of their child's illness is the effort to maintain emotional balance and seek appropriate support from the loved ones and relatives (Byra et al., 2021). In this regard, the results of another study show that communication among the mothers with newborns hospitalized in the NICU, their sharing of information and the emotional support they provide for each other contributes to positive orientation and further growth (Dhanoa & Singh, 2021). In other words, social interactions that are formed as a result of developing a disease or being near a patient may lead to useful changes in relationships where individuals may feel closer to others and, by gaining a better understanding of themselves and their lives, seek to establish strong relationships or change people's values and attitudes towards others. In the present study, the majority of the mothers had a higher tendency to social relationships, trying to cope and adapt to the stress caused by their newborn's hospitalization, which, in turn, may lead to higher growth in this dimension.

Although the experience of a child's severe disease is considered a stressful factor for parents, in the present study, mothers have grown in all dimensions after going through this unpleasant experience. Since PTG in the parents of NICU hospitalized newborns is highly correlated with parental psychological factors (Aftyka et al., 2020)، perceiving the stressful situation, personality factors and coping strategies (Dhanoa & Singh, 2021), it is recommended to conduct more studies on the other factors affecting PTG in these mothers.

One of the important limitations of this study is the time elapsed since the stressful event. Therefore, it is not clear how different mothers' experiences may be over a shorter or longer period of time. In addition, due to the need for a high number of samples in factor analysis, the number of the samples is also considered another limitation. This fact that some complications such as low birth weight or preterm labor could possibly impact mothers' responses, generalizing these findings to mothers of neonates with a history of hospitalization in the Neonatal Intensive Care Unit should be performed carefully. Moreover, it is suggested that future studies involve a diverse population sample.

## 5 CONCLUSION

In general, the results of this study showed that PTGI in the mothers with the experience of having NICU hospitalized newborns has appropriate psychometric properties and can be used as a valid and reliable scale in some research protocols and in different settings among these mothers.

## 6 RELEVANCE TO CLINICAL PRACTICE

This is because understanding positive psychological changes, identifying facilitators and using PTG dimensions to provide coping strategies can help nurses in planning and determining family‐centered care interventions to reduce the impact of the mental trauma caused by the newborn's hospitalization in parents, especially mothers, and to prevent the symptoms of PTSD. According to the dimensions of PTG and the obtained scores, in order to improve the use of individual abilities in the field of infant care and personal life, appreciation of life and striving for spiritual changes, efforts should be made with regard to the dimensions *communication with others* and *new opportunities*. In this case, the negative effect of such a stressful event can be turned into a valuable opportunity for mothers' growth and better experiences to increase their efficiency in care and parenting.

## CONFLICT OF INTEREST STATEMENT

None declared.

## Data Availability

Data are the responsibility of the author. These data can be provided upon request. The present study is approved by the ethics code IR.SBMU.PHARMACY.REC.1397.021 in Shahid Beheshti University of Medical Sciences.

## References

[nop21779-bib-0001] Aflakseir, A. , & Manafi, F. (2018). Posttraumatic growth and its relationship with cognitive emotion regulation strategies in patients with multiple sclerosis in shiraz, Iran. Practice in Clinical Psychology, 6(1), 57–62. 10.29252/nirp.jpcp.6.1.57

[nop21779-bib-0002] Aftyka, A. , Rozalska, I. , & Milanowska, J. (2020). Is post‐traumatic growth possible in the parents of former patients of neonatal intensive care units? Annals of Agricultural and Environmental Medicine, 27(1), 106–112. doi:10.26444/aaem/105800 32208588

[nop21779-bib-0003] Aftyka, A. , Rozalska‐Walaszek, I. , Rosa, W. , Rybojad, B. , & Karakuła‐Juchnowicz, H. (2017). Post‐traumatic growth in parents after infants' neonatal intensive care unit hospitalisation. Journal of Clinical Nursing, 26(5–6), 727–734. 10.1111/jocn.13518 27539892

[nop21779-bib-0004] Aftyka, A. , Rybojad, B. , Rosa, W. , Wróbel, A. , & Karakuła‐Juchnowicz, H. (2017). Risk factors for the development of post‐traumatic stress disorder and coping strategies in mothers and fathers following infant hospitalisation in the neonatal intensive care unit. Journal of Clinical Nursing, 26(23–24), 4436–4445. 10.1111/jocn.13773 28231614

[nop21779-bib-0005] Aslam, N. , & Kamal, A. (2019). Assessing positive changes among flood affected individuals: Translation and validation of posttraumatic growth inventory‐short form. Pakistan Journal of Medical Research, 58(2), 59–65.

[nop21779-bib-0006] Aydin, R. , & Kabukçuoğlu, K. (2020). The factor structure of the posttraumatic growth inventory in cancer patients in Turkey. Health & Social Care in the Community, 28(5), 1603–1610. 10.1111/hsc.12985 32342592

[nop21779-bib-0007] Barr, P. (2011). Posttraumatic growth in parents of infants hospitalized in a neonatal intensive care unit. Journal of Loss and Trauma, 16(2), 117–134. 10.1080/15325024.2010.519265

[nop21779-bib-0008] Boztepe, H. , Inci, F. , & Tanhan, F. (2015). Posttraumatic growth in mothers after infant admission to neonatal intensive care unit. Paediatria Croatica, 59(1), 14–18. doi:10.13112/PC.2015.3

[nop21779-bib-0009] Brelsford, G. M. , Doheny, K. K. , & Nestler, L. (2020). Parents' post‐traumatic growth and spirituality post‐neonatal intensive care unit discharge. Journal of Psychology and Theology, 48(1), 34–43. 10.1177/0091647119856468

[nop21779-bib-0010] Byra, S. , Zubrzycka, R. , & Wójtowicz, P. (2021). Positive orientation and posttraumatic growth in mothers of children with cystic fibrosis‐mediating role of coping strategies. Journal of Pediatric Nursing, 57, e1–e8. 10.1016/j.pedn.2020.09.009 32972807

[nop21779-bib-0011] Cadell, S. , Suarez, E. , & Hemsworth, D. (2015). Reliability and validity of a French version of the posttraumatic growth inventory. Open Journal of Medical Psychology, 4(2), 53–65. 10.4236/ojmp.2015.42006

[nop21779-bib-0012] Cheng, C. H. , Ho, S. M. , & Rochelle, T. L. (2017). Examining the psychometric properties of the Chinese post‐traumatic growth inventory for patients suffering from chronic diseases. Journal of Health Psychology, 22(7), 874–885. 10.1177/1359105315617330 26612723

[nop21779-bib-0013] Dhanoa, S. K. , & Singh, M. (2021). Predicting posttraumatic growth among primary caregivers of neonates with hyperbilirubinemia admitted in neonatal intensive care unit. International Journal of Psychological Studies, 13(1), 34–39. 10.5539/ijps.v13n1p34

[nop21779-bib-0014] García, F. E. , & Wlodarczyk, A. (2016). Psychometric properties of the posttraumatic growth inventory–short form among Chilean adults. Journal of Loss and Trauma, 21(4), 303–314. 10.1080/15325024.2015.1108788

[nop21779-bib-0015] Heidarzadeh, M. , Naseri, P. , Shamshiri, M. , Dadkhah, B. , Rassouli, M. , & Gholchin, M. (2017). Evaluating the factor structure of the Persian version of posttraumatic growth inventory in cancer patients. Asian Nursing Research, 11(3), 180–186. 10.1016/j.anr.2017.07.003 28991598

[nop21779-bib-0016] Heidarzadeh, M. , Rassouli, M. , Brant, J. M. , Mohammadi‐Shahbolaghi, F. , & Alavi‐Majd, H. (2018). Dimensions of posttraumatic growth in patients with cancer: A mixed method study. Cancer Nursing, 41(6), 441–449. 10.1097/NCC.0000000000000537 28806305

[nop21779-bib-0017] Ho, S. M. , Chan, C. L. , & Ho, R. T. (2004). Posttraumatic growth in Chinese cancer survivors. Psycho‐Oncology, 13(6), 377–389. 10.1002/pon.758 15188445

[nop21779-bib-0018] Keeley, J. W. , English, T. , Irons, J. , & Henslee, A. M. (2013). Investigating halo and ceiling effects in student evaluations of instruction. Educational and Psychological Measurement, 73(3), 440–457. 10.1177/0013164412475300

[nop21779-bib-0019] Kline, R. (2013). Exploratory and confirmatory factor analysis. In Y. Petscher & C. Schatschneider (Eds.), Applied quantitative analysis in the social sciences (pp. 171–207). Routledge.

[nop21779-bib-0020] Kline, R. B. (2015). Principles and practice of structural equation modeling. Guilford Publications.

[nop21779-bib-0021] Lamela, D. , Figueiredo, B. , Bastos, A. , & Martins, H. (2014). Psychometric properties of the Portuguese version of the posttraumatic growth inventory short form among divorced adults. European Journal of Psychological Assessment, 30(1), 3–14. 10.1027/1015-5759/a000161

[nop21779-bib-0022] Li, Y. , Cao, F. , Cao, D. , Wang, Q. , & Cui, N. (2012). Predictors of posttraumatic growth among parents of children undergoing inpatient corrective surgery for congenital disease. Journal of Pediatric Surgery, 47(11), 2011–2021. 10.1016/j.jpedsurg.2012.07.005 23163991

[nop21779-bib-0023] Morris, B. A. , Wilson, B. , & Chambers, S. K. (2013). Newfound compassion after prostate cancer: A psychometric evaluation of additional items in the posttraumatic growth inventory. Supportive Care in Cancer, 21(12), 3371–3378. 10.1007/s00520-013-1903-7 23928999

[nop21779-bib-0024] Osei‐Bonsu, P. E. , Weaver, T. L. , Eisen, S. V. , & Vander Wal, J. S. (2012). Posttraumatic growth inventory: Factor structure in the context of DSM‐IV traumatic events. ISRN Psychiatry, 2012, 937582. 10.5402/2012/937582 23738193 PMC3667636

[nop21779-bib-0025] Pajón, L. , Greco, A. M. , Pereda, N. , & Gallardo‐Pujol, D. (2020). Factor structure of the posttraumatic growth inventory in a Spanish sample of adult victims of interpersonal violence in childhood. Journal of Psychopathology and Clinical Psychology, 25(2), 101–110. 10.5944/rppc.26017

[nop21779-bib-0026] Palmer, G. A. , Graca, J. J. , & Occhietti, K. E. (2012). Confirmatory factor analysis of the posttraumatic growth inventory in a veteran sample with posttraumatic stress disorder. Journal of Loss and Trauma, 17(6), 545–556. 10.1080/15325024.2012.678779

[nop21779-bib-0027] Perez‐San‐Gregorio, M. A. , Martín‐Rodriguez, A. , Sanchez‐Martin, M. , Borda‐Mas, M. , Avargues‐Navarro, M. L. , Gomez‐Bravo, M. A. , & Conrad, R. (2018). Spanish adaptation and validation of the transplant effects questionnaire (TxEQ‐Spanish) in liver transplant recipients and its relationship to posttraumatic growth and quality of life. Frontiers in Psychiatry, 9, 148. 10.3389/fpsyt.2018.00148 29720952 PMC5915644

[nop21779-bib-0028] Rodriguez Rey, R. , Alonso Tapia, J. , Kassam‐Adams, N. , & Garrido Hernansaiz, H. (2016). The factor structure of the posttraumatic growth inventory in parents of critically ill children. Psicothema, 28(4), 495–503. 10.7334/psicothema2016.162 27776621

[nop21779-bib-0029] Shirinabadi Farahani, A. , Heidarzadeh, M. , Tajalli, S. , Ashrafizade, H. , Akbarpour, M. , Khaki, S. , Khademi, F. , Beikmirza, R. , Masoumpoor, A. , & Rassouli, M. (2021). Psychometric properties of the Farsi version of posttraumatic growth inventory for children‐revised in Iranian children with cancer. Asia‐Pacific Journal of Oncology Nursing, 8(3), 295–303. 10.4103/apjon.apjon-2093 33850963 PMC8030598

[nop21779-bib-0030] Silva, T. L. G. , Ramos, V. G. , Donat, J. C. , Oliveira, F. R. , Gauer, G. , & Kristensen, C. H. (2018). Psychometric properties of the posttraumatic growth inventory in a sample of Brazilian university students. Trends in Psychiatry and Psychotherapy, 40(4), 292–299. 10.1590/2237-6089-2017-0050 30570101

[nop21779-bib-0031] Taku, K. , Cann, A. , Calhoun, L. G. , & Tedeschi, R. G. (2008). The factor structure of the posttraumatic growth inventory: A comparison of five models using confirmatory factor analysis. Journal of Traumatic Stress, 21(2), 158–164. 10.1002/jts.20305 18404631

[nop21779-bib-0032] Tedeschi, R. G. , & Calhoun, L. G. (1996). The posttraumatic growth inventory: Measuring the positive legacy of trauma. Journal of Traumatic Stress, 9(3), 455–471. 10.1007/BF02103658 8827649

[nop21779-bib-0033] Walsh, D. M. , Groarke, A. M. , Morrison, T. G. , Durkan, G. , Rogers, E. , & Sullivan, F. J. (2018). Measuring a new facet of post traumatic growth: Development of a scale of physical post traumatic growth in men with prostate cancer. PLoS One, 13(4), e0195992. 10.1371/journal.pone.0195992 29702656 PMC5922578

[nop21779-bib-0034] Wilson, C. , & Cook, C. (2018). Ambiguous loss and post‐traumatic growth: Experiences of mothers whose school‐aged children were born extremely prematurely. Journal of Clinical Nursing, 27(7–8), e1627–e1639. 10.1111/jocn.14319 29495088

